# Formal language assessment in low-educated healthy subjects

**DOI:** 10.1590/1980-57642018dn12-030009

**Published:** 2018

**Authors:** Daniela Aiko Akashi, Karin Zazo Ortiz

**Affiliations:** 1 Speech Pathologist, Department of Speech, Language and Hearing Sciences, Universidade Federal de São Paulo (UNIFESP), São Paulo, SP, Brazil.; 2 PhD, Speech Pathologist, Associate Professor, Department of Speech, Language and Hearing Sciences, Universidade Federal de São Paulo (UNIFESP), São Paulo, SP, Brazil.

**Keywords:** language, educational status, neuropsychological tests, language disorders, linguagem, testes neuropsicológicos, distúrbios de linguagem, escolaridade

## Abstract

Although many studies have shown the influence of education on cognition, the impact of low education on the various cognitive functions appears to differ. The hypothesis of the present study is that, with regards to language, the use of parameters derived from populations with 5-8 years of education leads to false-positive results.

**Objective:**

to determine the influence of low education on the language tasks assessed by the MTL-Brazil Battery

**Methods:**

30 healthy adults with 2-4 years of education were submitted to the MTL-Br Battery, comprising 22 subtests. The data were submitted to descriptive statistical analysis for each subtest and Z-scores were then calculated based on the parameters of a population with 5-8 years of education. All participants would be considered impaired if the Battery had been applied according to published normative criteria for a population with 5-8 years of education.

**Results:**

Separate analysis revealed that published scores for 17 out of the 22 Battery tasks were inappropriate for a population with 2-4 years of education.

**Conclusion:**

Education was found to effect performance for each of the language abilities differently. In addition, the study results can be applied to language assessments of individuals with 1-4 years of education using the MTL-Br battery, since this is the only language test for adults available in Brazil, and for which there are no normative data for low-educated subjects.

## Resumo

As funções cognitivas, dentre elas a linguagem, sofrem a interferência da escolaridade. A hipótese deste estudo é que mesmo o uso de parametros obtidos em populações de 5 a 8 anos de escolaridade pode levar a falsos-positivos em populações com escolaridade de 2 a 4 anos de estudo.

**Objetivo:**

Verificar a influência da baixa escolaridade nas tarefas linguísticas da bateria MTL-Br e obter dados que possam ser utilizados como parâmetros clínicos nas avaliações de indivíduos cérebro-lesados de baixa grau de escolaridade.

**Métodos:**

Trata-se de um estudo prospectivo, em que foram avaliados 30 indivíduos normais de baixa escolaridade, de dois a quatro anos de estudo, com idade entre 19 e 60 anos, de ambos os sexos. Os indivíduos foram submetidos ao Mini-Exame do Estado Mental e Teste do Desenho do Relógio para fins de rastreio cognitivo.

**Resultados:**

Todos os indivíduos que obtiveram escores compatíveis com os parâmetros de normalidade nestes dois testes foram submetidos à bateria MTL-Br. A avaliação seguiu estritamente as normas publicadas no instrumento original e o registro foi realizado nas folhas da própria bateria.

**Conclusão:**

No presente estudo evidenciamos a influência da escolaridade em tarefas linguísticas. Além disso, foi possível obter dados que poderão ser utilizados como parâmetros clínicos nas avaliações de indivíduos cérebro-lesados de baixa escolaridade.

Various cognitive functions, including language, are affected by education.^1^^-^ 4 Understanding the role of education in cognitive functioning is of fundamental importance because learning to read and write, together with all experiences gained at school, is believed to influence brain organization^5^ and performance on neuropsychological tasks and represent a protective factor against neurological diseases and decline in cognitive functions.^6^ In addition, the application of tests for which no education-related parameters exist can lead to false-positive results in low-educated individuals.

In Brazil, the first tests specifically for assessing language in adults were studied in the 1990s,^7^^,^ 8 while the first battery^9^ with adequate psychometric data^10^^,^ 11 for assessing language was published only in 2016. Given that such tests are a fundamental element in neuropsychological assessment, knowledge about the influence of education on language assessment protocols in use is important. This is especially true when used in developing countries such as Brazil, which is socioculturally diverse in terms of schools (public or private) and educational level attained by the general population.

Previous studies involving specific language tests have shown the influence of education on performance of neurologically healthy individuals in many language tasks,^2^^,^ 8 ^,^ 9 ^,^ 12 including both written and oral ones.

Studies investigating the influence of education on cognitive functions assessed by different tests have stratified educational into bands. The most common strata used are illiterate, 1-4 years education, 5-8 years, 8-12 years and over 12 years of education, although no consensus on this division exists. These studies have tended to find greater homogeneity in cognitive behavior among individuals with 8 or more years of education.^1^^,^ 3 ^,^ 4 ^,^ 13 The same effect can be observed on language tests, although tasks that are highly dependent on the lexical buffer, such as oral and written naming and verbal fluency tests, appear to differ.^2^^,^ 12

Although many studies demonstrate the influence of education on cognitive development,^1^^-^ 4 ^,^ 8 ^,^ 9 ^,^ 12 ^,^ 13 the impact of low education on the various cognitive functions appears to differ. In a previous study assessing several cognitive domains of individuals with 0-22 years of formal education, the authors noted that the education effect was non-linear: significant differences were evident between groups with 0-3 versus 10-22 years of formal education, whereas the same was not observed between groups with 4-9 and 10-22 years of formal education. In addition, no difference in attention and orientation domains were found between individuals with ≥4 years of education and those with ≤9 years of education, who had similar performance for memory.^14^ This data is very important since it suggests that formal education does not impact all the cognitive domains in the same way. A number of studies investigating different cognitive functions reveal statistically significant disparities between groups with different levels of education on linguistic tasks such as oral comprehension, reading, graphical comprehension, naming, lexical availability, dictation and written naming of actions,^2^^,^ 8 ^,^ 12 as well as on language tasks involving semantic knowledge, judgement of traits and naming, besides tasks of calculus,^3^ memory^1^ and orofacial praxis.^15^ Depending on the tasks or tests employed, some studies have found differences only between illiterate subjects and educated individuals, irrespective of level of education,^13^ whereas others have reported disparities across all^3^ or some educational levels.^16^ With regard to language, given the complexity and number of different language tasks needed to assess this ability, performance on different language tasks according to education are highly disparate. The hypothesis of the present study is that, with regards to language, the impact of low education is so significant that the use of parameters derived from populations with 5-8 years of education leads to false-positive results.

Therefore, the primary objectives of this study were to determine the influence of low education on the language tasks assessed by the MTL-Brazil Battery and to compare language performance of a low-educated population with 2-4 years of education against that of a population with 5-8 years education to ascertain which language abilities are most susceptible to false-positive results. The secondary objective of the study was to obtain data for use as clinical parameters in assessments of brain-injured low-educated individuals using the MTL-Br battery, given that no normative data for use in low-educated subjects are available.

## METHODS

This prospective pilot study was approved by the Research Ethics Committee (permit 1219/2017). All study participants agreed to take part by signing the Free and Informed Consent form.

The study inclusion criteria were: age 19-59 years, education ≥1 year and normal performance on cognitive screening tests, attaining score ≥18 points on the Mini-Mental State Exam^4^ and ≥7 points on the Clock Drawing Test.^17^

The exclusion criteria were: illiteracy, history of previous or current learning and/or language difficulties, diagnosis or history of visual or hearing impairments which could prevent test completion, history of prior or current psychiatric or neurologic disorders, use of legal or illegal psychotropic drugs (except atypical neuroleptics) and alcoholism, according to the inclusion criteria for neuropsychology studies described in MOANS (Mayo Clinic’s Older Americans Normative Studies).^18^ This information was collected by applying a questionnaire. In order to better characterize the study participants and given that reading and writing habits can affect cognitive performance,^12^ the questionnaire responses for these habits were analyzed. Participants indicated the frequency of reading and writing for different materials, with scores attributed as follows: 4 points - “every day”, 3 points - “several days per week”, 2 points - “once per week”, 1 point - “rarely” and 0 points - “never”. Reading habit score ranged from 0 to 16 points and writing habit from 0 to 12 points.

All participants that met the inclusion criteria were submitted to the Brazilian version of the Montreal-Toulouse Language Assessment Battery, Brazilian version (MTL-Br).^9^ The test was applied individually by the same examiner in a quiet room, with application time limited to 1 hour, according to the instructions contained in the application manual. The test sheets from the MTL-Brazil Battery were used: application booklets I and II. The test comprises 22 subtests as outlined below:

1. Structured interview: includes 13 open-ended questions. Total score ranges from 0 to 26 points.
2. Automatic speech: assesses the ability to evoke automatisms, evaluated for form and content. Total score ranges from 0 to 6 points.
3. Oral comprehension: measures auditory comprehension of words and phrases. Total score ranges from 0 to 19 points, with 1 point given for each correct answer.
4. Oral narrative discourse: evaluates the ability to tell a story from visual inputs. On this test, the total number of words produced and the production of a narrative are analyzed. One point is given for each of these elements present in the discourse produced.
5. Written comprehension: assesses the written comprehension of words and phrases. One point is given for each correct answer, with total task score ranging from 0 to 13 points.
6. Copying: a sentence must be copied while changing the allographic form. Maximum task score is 8 points.
7. Written dictation: assesses the participant’s ability to write dictated words and phrases. One point is given for each word written correctly, with total score ranging from 0 to 22 points.
8. Repetition: measures the ability to reproduce the auditory stimuli provided. One point is given for each correctly repeated item, yielding a score of 0 to 33 points.
9. Reading aloud: assesses reading of words and phrases. Each correctly read item is scored, with total score ranging from 0 to 33 points.
10. Semantic verbal fluency: evaluates the ability for lexical production of words from the animals semantic category within 90 seconds. One point is given for each correctly produced word.
11. Non-verbal praxis: assesses the ability to produce non-verbal praxic gestures. Score on the task ranges from 0 to 24 points.
12. Naming: measures lexical access using pictures that refer to nouns and verbs. Score on this task ranges from 0 to 30 points.
13. Object manipulation by verbal command: assesses the ability to understand simple and complex verbal commands. This task is scored from 0 to 16 points.
14. Phonological verbal fluency: evaluates the number of words produced beginning with the letter M within 90 seconds. A point is given for each word produced.
15. Body part recognition and left-right orientation: assesses recognition of parts of the body and laterality orientation. This task is scored from 0 to 8 points.
16. Written naming: each correctly written word scores two points. Score ranges from 0 to 30 points.
17. Oral text comprehension: assesses comprehension of simple narrative text. Scores on this subtest range from 0 to 9 points.
18. Number dictation: assesses the ability to transcode six numbers from auditory stimuli to written form. Score ranges from 0 to 6 points.
19. Reading of numbers: assesses the ability to recognize six arabic numerals and reproduce them orally. Score ranges from 0 to 6 points.
20. Written narrative discourse: involves the ability to write a story from visual input. On this task, the number of words and the presence of a narrative are analyzed.
21. Written text comprehension: assesses the ability to understand a written text. Score on this task ranges from 0 to 9 points.
22. Numerical calculation: evaluates the ability to perform mathematical calculations and solve mathematical problems. Each subtest - mental and written calculations - is scored from 0 to 6 points.

After applying the MTL Battery in 30 low-educated individuals, the data were submitted to descriptive statistical analysis to determine the mean, standard deviation, lower and upper limits for each subtest of the MTL-Brazil Battery.

To check whether a diagnostic error would be incurred by applying the Z-score using the normalization data for the MTL-Brazil Battery from individuals with 5-8 years education, the Z-score was calculated for each subtest in all study participants assessed.

The use of a cut-off point of Z ≤-1.5 provides an index representative of neurological impairment^19^. Therefore, four classifications were used to analyze the Z-score:

- Z-score of -1.0 to -1.5: indicates possible neuropsychological impairment;
- Z-score ≤-1.5: indicates presence of mild neuropsychological impairment;
- Z-score of -1.6 to -2.0: indicates moderate-to-severe impairment;
- Z-score ≤-2.0: suggests presence of very severe impairment.

## RESULTS

### Sample characteristics

A total of 32 healthy adults took part in this study. Of this group, 2 were subsequently excluded for not attaining normal scores on the cognitive screening tests. Thus, a final study sample of 30 healthy adults was assessed, comprising 24 (80%) women and 6 (20%) men, aged 31-59 years (mean=44.3 years, SD=8.8 years).

Education ranged from 2-4 years, where 18 (60%) individuals had 4 years of formal education, 9 (30%) had 3 years and 3 (10%) had 2 years of education.

Among the sample, 19 (63.3%) participants had low frequency of reading and writing habits and 11 (36.7%) high frequency. Mean total score was 11.2 points (standard deviation 5.6).

Mean score on the Mini-Mental State Exam was 26.9 points (SD=2), range 19-30 points, and mean score on the Clock Drawing Test was 9 points (SD=1.4).

The results of healthy low-educated subjects on the MTL-Brazil Battery are presented in Table 1.

**Table 1:** Performance of healthy low–educated individuals on language tasks of the MTL – Brazil Battery.

Tasks	Mean	Median	Minimum	Maximum	Standard deviation
Structured Interview	25.4	26.0	21	26	1.0
Automatic Speech – Form	5.8	6.0	4	6	0.6
Automatic Speech – Content	5.7	6.0	4	6	0.6
Oral Comprehension – Words	4.9	5.0	4	5	0.18
Oral Comprehension – Phrases	11.8	12.0	9	14	1.3
Oral Comprehension – Total	16.8	17.0	14	19	1.3
Oral Narrative Discourse – Number of Words	40.7	35.5	14	87	17.4
Oral Narrative Discourse – Narration of Story	1.9	2.0	1	2	0.3
Written Comprehension – Words	4.9	5.0	3	5	0.3
Written Comprehension – Phrases	6.1	6.0	3	8	1.5
Written Comprehension – Total	11.1	11.0	6	13	1.7
Copying	7.7	8.0	5	8	0.7
Written Dictation	14.3	14.5	2	22	5.3
Repetition – Words	10.8	11.0	9	11	0.5
Repetition – Phrases	21.9	22.0	21	22	0.3
Repetition – Total	32.7	33.0	30	33	0.6
Reading Aloud – Words	8.9	9.0	2	12	2.4
Reading Aloud – Phrases	20.2	21.0	16	21	1.4
Reading Aloud – Total	29.2	30	29	33	3.4
Semantic Verbal Fluency	16.5	16.0	7	25	4.2
Non-Verbal Praxis	23.6	24.0	20	24	1.0
Naming – Nouns	21.5	22.0	12	24	2.8
Naming – Verbs	5.4	6.0	0	6	1.3
Naming – Total	27.0	28.0	15	30	3.4
Object Manipulation by Verbal Command	15.9	16.0	15	16	0.1
Phonological Verbal Fluency	9.9	6.0	2	19	4.3
Left-Right Orientation	3.9	4.0	2	4	0.3
Body Part Recognition	3.9	4.0	2	4	0.3
Body Part Recognition and L-R Orientation – Total	7.8	8.0	4	8	0.7
Written Naming – Words	16.1	13.5	0	24	5.2
Written Naming – Verbs	3.3	2.0	0	6	2.1
Written Naming – Total	19.5	15.5	0	30	6.3
Oral Text Comprehension	5.5	6.0	0	9	2.5
Number Dictation	5.2	5.0	4	6	0.7
Reading of Numbers	5.2	5.0	4	6	0.6
Written Narrative Discourse – Number of Words	18.7	18.0	4	42	10.8
Written Narrative Discourse – Analysis of Production-Narration of Story	1.8	2.0	1	2	0.4
Written Text Comprehension	6.6	7.0	2	9	2.3
Numerical Calculation – Mental	3.0	3.0	0	6	1.6
Numerical Calculation – Written	2.8	3.0	0	6	2.0
Numerical Calculation – Total	5.8	6.0	0	12	3.2

As there are still no normative data available on low-educated subjects for the MTL-Br battery, in order to determine the number of individuals that could be wrongly diagnosed with impairment, Z-scores were calculated for all scores of each individual assessed, on all battery tasks, using the normative parameters of individuals with 5-8 years education as a reference. The language tasks on which performance differences were evident in the group with 2-4 years of education, together with the percentage of individuals whose Z-score indicated impairment, are presented in Table 2.

**Table 2:** Percentage of healthy low-educated (2-4 years education) individuals whose performance indicated impairment based on Z-score calculation, using normative data for a population with 5-8 years education as a reference. Language tasks that showed differences between groups with 1-4 and 5-8 years education.

Tasks	Mild (%)	Moderate (%)	Severe (%)	Total (N)	Total (%)
Total Automatic Speech – Form	0	0	17	5	17
Oral Comprehension – Phrases	10	10	27	14	47
Oral Comprehension – Total	20	0	27	14	47
Oral Narrative Discourse – Number of Words	17	3	0	6	20
Oral Narrative Discourse – Narration of Story	0	87	13	30	100
Written Comprehension – Words	0	0	3	1	3
Written Comprehension – Phrases	3	0	40	13	43
Written Comprehension – Total	20	0	40	18	60
Copying	0	0	20	6	20
Written Dictation	13	7	37	17	57
Repetition – Words	3	0	10	4	13
Repetition – Phrases	0	0	10	3	10
Repetition – Total	0	0	13	4	13
Reading Words Aloud	10	7	43	18	60
Reading Aloud – Phrases	17	0	30	14	47
Reading Aloud	17	7	47	21	71
Semantic Verbal Fluency	30	3	3	11	36
Non-Verbal Praxis	0	0	17	5	17
Naming – Nouns	0	23	27	15	50
Naming – Verbs	3	3	17	7	23
Naming – Total	13	3	40	17	56
Object Manipulation by Verbal Command	0	0	3	1	3
Phonological Verbal Fluency	30	3	7	12	40
Left-Right Orientation	0	0	3	1	3
Body Part Recognition	0	0	3	1	3
Body Part Recognition and L-R Orientation – Total	0	0	3	1	3
Written Naming – Words	13	10	43	20	66
Written Naming – Verbs	7	0	43	15	50
Written Naming – Total	13	13	47	22	73
Oral Text Comprehension	7	3	17	8	27
Number Dictation	13	0	57	21	70
Reading of Numbers	0	23	47	21	70
Written Narrative Discourse – Number of Words	30	0	0	9	30
Witten Narrative Discourse – Analysis of Production-Narration of Story	43	23	0	20	66
Written Text Comprehension	3	10	27	12	40
Numerical Calculation – Mental	7	3	27	11	37
Numerical Calculation – Written	17	0	33	15	50
Numerical Calculation – Total	17	7	30	16	54

## DISCUSSION

The most important finding of this study was that education influenced performance on the language tasks in different ways. The results also highlighted that this complex cognitive function requires in-depth study to determine how each year of education affects the language profile, as reflected in performance on the different language tasks, where the use of inadequate parameters can lead to a high rate of false-positive results. This finding is discussed further below.

Regarding the sample characteristics, the high number of individuals with 3-4 years education (90% of total individuals assessed) showed that, despite the predominance of years of education closer to the parameters used in the Z-score calculation (Table 2), the effect of education on the tasks of the MTL-Brazil Battery was evident. In addition, the casuistic comprised predominantly participants who had low frequency of reading and writing habits, a factor influencing cognitive development that can contribute to performance on neuropsychological and language tasks.^20^

The structured interview proved a simple task even for low-educated subjects (Table 1). The questions generally refer to everyday situations whose familiar context aids understanding.

The results in Table 2 show that, on the oral comprehension task, abnormal Z-scores occurred only on the phrase comprehension task in some individuals. Moreover, there was a greater presence of errors on complex phrases, which require attention to grammatical structure and short-term retention of the phrase, relying on working memory for comprehension.^2^^,^ 12 ^,^ 21 The same difficulty for this type of syntactic processing has been observed in previous studies using non-canonical structures, particularly passive voice.^22^ Operations involving syntactic analysis are introduced later in the school curriculum and tasks involving working memory can be influenced by education.^23^^,^ 24 The same holds true for processing of written sentences. In this case, there is the additional need for the ability to decode graphemes and lexemes for effective reading comprehension^2^ In addition, line drawings are more challenging for low-educated individuals because visual analysis of two-dimensional representations are tasks that require more years of schooling^25^ and the fact that drawings from the test are in black and white and that those for complex phrases involve a greater level of detail may have further hampered recognition.^26^^,^ 27 This aspect of visual processing may have affected all tasks relying on this type of analysis, such as oral and written naming.

With regard to the oral and written narrative tasks, the descriptive task predominated. This characteristic is due to the fact that the narrative composition requires coordination of the different cognitive and communication abilities, such as organization of ideas, inter-relationships between characters, and so on. The written discourse was more difficult than the oral one, even though the plates have the same level of semantic complexity. This is due to the fact that the abilities involved in the writing process develop and improve during the years of formal study^28^ and the formal pressures demanded by the written code make this type of narrative discourse structurally more complex than speech.^29^

On the written dictation task, there was a predominance of errors in writing words that rely on the lexical route, which require the presence of the stimulus in the logographic lexicon. In addition, low-educated individuals had difficulty choosing the grapheme to be used in words with phonemes that had different graphemic representations^26^ and the frequency of errors in written words by the lexical route is due to the fact that schools focus on reading by the perilexical pathway in the first years of education.

Worse performance was observed on the repetition task compared to previous studies.^22^^,^ 26 This disparity is probably due to the presence of pseudowords, whose repetition depends on the phonological route responsible for phonemic coding,^30^ typically underdeveloped in illiterate or low-educated subjects,^31^ leading to lexicalization errors.^32^

With regard to semantic verbal fluency, previous studies have found an influence of different educational levels,^2^^,^ 8 ^,^ 22 explained by the fact that formal learning facilitates the organization of semantic categories and subgroups.^33^ There is a greater education effect on phonological verbal fluency tasks, owing to the association between phonological awareness and literacy.^33^

The low performance of the sample on text comprehension might be related to working memory,^23^^,^ 25 given that during comprehension, construction of a mental representation occurs that must remain active to understand information yet to be processed.^34^

The individuals performed similarly on the number dictation and reading tests (Table 1). A previous study^3^ also showed that tasks involving calculations and numerical processing are strongly dependent on educational level.

On the mental and written numerical calculation tasks, greater difficulties in solving mathematical problems and performing multiplication and division operations were evident. This finding is due the manner in which the rules for these less systematically studied operations are learned and retained. For example, the rules for multiplication are generally memorized or trained by chanting rhymes^3^ and feature later in the school curriculum. Lastly, the effect of education was also evident on non-verbal praxis, a finding reported in a previous study^15^.
